# Weaning challenges and the effectiveness of nurse-tailored interventions in supporting dietary transition among infants: A study in urban India

**DOI:** 10.6026/973206300211956

**Published:** 2025-07-31

**Authors:** Muthupetchi Ramachandran, Shankar Shanmugam Rajendran, Vanitha Narayanasamy Naidu, Anitha Parasuraman, Gnana Malar Periyakaruppan

**Affiliations:** 1 Department of Community Health Nursing, College of Nursing, Madras Medical College, The Tamil Nadu Dr. MGR Medical University, Chennai, Tamil Nadu, India; 2 Department of Child Health Nursing, College of Nursing, Madras Medical College, The Tamil Nadu Dr. MGR Medical University, Chennai, Tamil Nadu, India

**Keywords:** Breastfeeding, weaning challenges, dietary transition, primary care providers, mixed study

## Abstract

Weaning challenges and the impact of a nurse-tailored intervention on dietary transitions among primary care providers of infants is of interest. A mixed-methods design was used, combining qualitative interviews with five providers and a quasi-experimental approach with 60 participants. The intervention significantly improved knowledge (40.15%) and practices (40.40%) in the experimental group. Thematic analysis showed the importance of medical advice, family influence and digital platforms in weaning guidance. Thus, nurse-tailored interventions can enhance knowledge and practices related to weaning in urban Indian settings, emphasizing the need for targeted educational programs.

## Background:

Breastfeeding provides essential immune-protective antibodies, long-chain fatty acids like DHA and vital amino acids, while reducing maternal risks for breast and ovarian cancers [1, 2]. However, exclusive breastfeeding (EBF) remains insufficient globally. The 2023 Global Breastfeeding Scorecard reports a slight increase to 48% EBF, nearing the World Health Assembly's 50% target. In India, the NFHS-5 shows a 63.7% EBF rate for infants less than six months, an improvement from 54.9% in NFHS-4; however, only 41.8% of mothers initiate breastfeeding within the first hour post-partum [3, 4]. After six months, breast milk alone is insufficient for infants' nutritional needs, necessitating the introduction of complementary foods for growth and neurodevelopment [5]. Primiparous and employed mothers face significant stress due to insufficient maternity leave, rigid schedules and lack of lactation facilities, leading to early formula supplementation [6, 7]. An early return to work often accelerates weaning, causing additional mental stress for both mother and child [8, 9]. Paid leave, flexible schedules and on-site lactation support are essential policy instruments [10]. Knowledge gaps further complicate the weaning process. In Saudi Arabia, only 35.1% of mothers have adequate breastfeeding knowledge, often relying on peer advice instead of healthcare professionals [11]. Similarly, Sudanese women with lower education and resources face greater challenges [12]. In India, 9.9% of employed mothers adhere to the recommended six months of EBF, while 48.1% introduce starch-based solids early and 28.2% rely on commercial foods for snacks [13]. Nurse-led educational programs have proven effective in bridging these gaps, with a Saudi initiative increasing post-test knowledge to 82.1% and appropriate practices to 80.7% [14]. Additionally, nurse counselling addressing psychosocial challenges improves coping during lactation [15]. Workplace constraints, cultural beliefs and psychological stress hinder effective weaning, but evidence suggests that a nurse-driven curriculum can improve knowledge by 30% and practice by 25% [16]. Therefore, it is of interest to describe the key barriers and effective strategies for supporting the transition from exclusive breastfeeding to complementary feeding, with a focus on culturally relevant nursing interventions in urban India.

## Materials and Methods:

This study utilised a mixed-method, Exploratory sequential design. A phenomenological design was employed for the qualitative component to explain the lived experiences of five primary care providers. The quantitative component used a quasi-experimental, non-randomized control group design with 60 participants, with 30 to the study group and 30 to the control group. Non-probability purposive sampling was employed for the qualitative component, whereas convenience sampling was utilised for the quantitative part. The nurse-tailored interventions encompassed organised instruction on newborn nutrition, feeding methodologies and a culinary demonstration. Preliminary and subsequent assessments were conducted to evaluate alterations in knowledge and practices. The tools used were a sociodemographic questionnaire, an unstructured interview schedule, a knowledge questionnaire consisting of 20 multiple-choice questions and a 10-item practice checklist. Validity and reliability were confirmed through content validity, a pilot study and internal consistency assessment. The research was conducted over four weeks in an urban area of Chennai, with ethical approval obtained from the Institutional Ethics Committee, Madras Medical College and Chennai (IEC-MMC/Approval/46112024). Post test was conducted after 21 days. Quantitative data were analysed with SPSS v26 employing t-tests and chi-square tests, whereas qualitative data were subjected to thematic analysis. Throughout the study, credibility, rigour, trustworthiness and triangulation were maintained. Data saturation was obtained and human rights safeguards were meticulously adhered to.

## Results:

This study aimed to explore the weaning challenges and the effect of nurse-tailored intervention on dietary transition among primary care providers of infants. The mean age of the participants was 34.8 ± 8.8 years. In the experimental group, most of the primary care providers were grandparents (50.00%), while parents predominated in the control group (60.00%). High school education was the most common level, 36.66% in the experimental group and 43.33% in the control group. Homemakers were the majority of participants (60.00% and 70.00%, respectively). Family income mostly ranged from Rs. 20,001-30,000 in the experimental group and Rs. 30,001-40,000 in the control group. Most households had one child. All participants were urban residents. The majority had not participated in any nutrition programs. Infants were mostly aged 10-12 months and Hinduism was the most common religion (53.33%).Thematic analysis showed the following themes and subthemes.

## Initial stages:

## Initiation of weaning:

Primary Care providers initiated weaning mainly at six months, guided by health care professionals. Timing varied slightly, showing awareness of infant readiness and the value of professional advice during early feeding transitions. "The doctor told me to come till 6 months, so after that..."- participant 3.

## Bodily reactions:

Infants often experience vomiting or diarrhea when introduced to new foods. These reactions emphasized the need for careful, individualized approaches and underscored the importance of observation and selecting appropriate foods. "Are you asking about the reactions? The baby had diarrhea". - Participant 1.

## Advice:

## Hospital advice:

Medical professionals played a key role in guiding weaning practices. Though caregivers trusted hospital staff, conflicting information with traditional norms occasionally led to confusion and hesitation in feeding choices. "For my elder daughter, only after the completion of 6 months, I started weaning. So, for them also, after 6 months, I started weaning. I also took the advice of the doctor and the advice of the pediatrician". - Participant 2.

## Near ones:

Family and neighbours influenced the primary care providers with culturally rooted advice. Despite occasional misinformation, their input created a strong support system and offered practical tips drawn from lived experiences. "No, whatever my mother has followed, I followed the same". - Participant 4.

## Internet:

Digital platforms like YouTube and Google were widely used for weaning guidance. Primary care providers appreciated the accessibility, though distinguishing reliable advice from misinformation required digital awareness and discernment. "Yes, they gave me information and apart from that, if I have doubts, I use Google, then watch videos on YouTube" - participant 5.

## Gradual:

## Little amount:

Primary care providers practiced gradual food introduction, starting with small quantities. This cautious approach helped infants adjust safely, preventing adverse reactions and building tolerance to a variety of foods over time. "Earlier she didn't eat properly, but now when I started giving little by little, she got used to it. I give all the food mixed..." - participant 3.

## Level of knowledge and practice on dietary transition:

Before the intervention, both groups had similar levels of knowledge and practice. In the experimental group, 63.33% showed inadequate knowledge and low practice, while in the control group, 60% experienced similar deficiencies (Table 1 - see PDF). There was no significant difference between them (p > 0.05). After the nurse-tailored intervention, the experimental group showed notable improvement (Table 2 - see PDF), with about 76.67% having adequate knowledge and 80% demonstrating good practice. In contrast, the control group continued to have lower levels of knowledge and practice. Chi-square tests revealed a significant difference (p = 0.001), indicating that the nurse-tailored interventions helped the experimental group to learn and perform better.

## Effectiveness of nurse-tailored intervention on knowledge and practice on dietary transition:

The experimental group demonstrated significant improvement following the nurse-tailored intervention. The average knowledge scores increased from 8.00 (40.00%) to 16.03 (80.15%), representing a 40.15% improvement. The average practice scores increased from 4.13 (41.30%) to 8.17 (81.70%), representing a 40.40% improvement. On the other hand, the control group saw just little improvements: knowledge went up by 2.85% and practice by 4.30%. The results show that the intervention was effective in helping the people in the experimental group learn more about and transition to baby food more successfully.

## Association with the socio-demographic variables:

The study found a significant link between primary care provider's age (χ^2^ = 6.99, p = 0.03), education (χ^2^ = 9.19, p = 0.05) and number of children (χ^2^ = 5.18, p = 0.02) with knowledge. Older primary care providers and those with higher education had better knowledge. For practice, significant associations were seen with caregiver's age (χ^2^ = 6.77, p = 0.05), relationship to the infant (χ^2^ = 5.99, p = 0.05) and number of children (χ^2^ = 4.32, p = 0.05). Other factors showed no significant association.

## Discussion:

The present study explored weaning challenges through the experiences of primary care providers, revealing several key themes and subthemes that supported with the existing literature. The Initial Stages theme highlighted the role of Initiation, with primary care providers often transitioning to new feeding practices based on their healthcare advice, indicating informed decision-making. This aligns with the findings of Ghada Mohammad Abu Shosha (2020), who found that mothers in Jordan also experienced difficulties and uncertainties during weaning, largely guided by medical advice [17]. In the Bodily Reactions subtheme, many mothers reported digestive issues like vomiting and diarrhea after introducing new foods, which highlighted the need for personalized care. This was support the findings of Fikadu *et al.* (2024), who similarly found that primipara mothers experienced both challenges and emotional impacts during weaning [18]. In the Gradual theme, Hospital advice was an important factor, with medical professionals playing a substantial role in shaping feeding practices. Similarly, near ones emphasized the impact of family advice, especially from grandmothers. This aligns with the insights of Abu Shosha (2020), who emphasized the impact of various cultural influences on mothers' decisions to wean their children [17]. Internet use was prevalent, with mothers often seeking guidance from platforms like YouTube. This finding aligns with a study by Fikadu *et al.* (2023), in which mothers utilized multiple sources of information, including online platforms, to support their weaning practices [18]. Similarly, the study assessed the effectiveness of a nurse-tailored intervention in improving primary care providers' knowledge and practices on weaning. The experimental group demonstrated significant improvements in both knowledge and practice scores, with findings supporting the positive impact of educational interventions, as seen in studies Amer *et al.* (2023) [19]. Demographic factors such as age, education level and number of children were found to be significantly influencing knowledge and practice scores in the experimental group. This supports the work of Patel *et al.* (2021) and Madhan *et al.* (2025), who observed a similar association between maternal knowledge and socio-demographic factors [20, 21].

## Conclusion:

Working mothers struggle with breastfeeding and weaning due to a lack of information and challenges at work and home. Primary care providers showed significant improvement in knowledge and practices with training. Thus, the importance of empowering nurses to provide essential guidance in the community for better infant feeding and health is shown.
